# Changes in Serum Prealbumin and Incision Complications Following Spinal Tuberculosis Surgery: A Preliminary Study

**DOI:** 10.1111/os.12896

**Published:** 2021-02-11

**Authors:** Yu‐cheng Bao, Ming Yu, Liang Tang, Hua‐ying Ning, Wen‐long Zhang

**Affiliations:** ^1^ Department of Orthopaedics, Tianjin Haihe Hospital, China Key Research Laboratory for Infectious Disease Prevention for State Administration of Traditional Chinese Medicine Tianjin University Tianjin China

**Keywords:** Debridement, Incision complications, Nutritional status, Serum prealbumin, Spinal tuberculosis

## Abstract

**Objectives:**

To explore the trend of changes in the serum prealbumin (PA) level in patients with spinal tuberculosis during the perioperative period and its relationship with postoperative incision complications.

**Methods:**

A retrospective study was performed by enrolling 162 patients (82 men and 80 women) with spinal tuberculosis who had been admitted to the Tianjin Haihe Hospital from June 2013 to June 2017. The included patients were then assigned to the elderly group (≥65 years of age, *n* = 35) and the non‐elderly group (<65 years of age, *n* = 127). The chemotherapy regimen was 3HREZ/9HRE, in combination with nutritional support for 3–4 weeks, as well as one‐stage debridement and (or) bone graft fusion and internal fixation. The serum PA levels of patients with spinal tuberculosis at admission, prior to surgery, and at 2 and 4 weeks after surgery were collected, and incision healing and sinus formation were observed for 3 months. Changes in serum PA levels of all patients at different time points were observed using one‐way analysis of variance. Pairwise comparison at different time points was performed using the least significant difference method and comparison of serum PA levels between different groups at the same time points was subjected to *t*‐test. The χ^2^‐test was used for comparison of the incidence of incision complications between different groups and between different subgroups based on different PA levels.

**Results:**

There was a gradual increased trend in the PA level from admission to 4 weeks after surgery in all patients [(0.14 ± 0.03) g/L < (0.16 ± 0.04)g/L < (0.22 ± 0.04) g/L < (0.25 ± 0.04) g/L]. The increase in the non‐elderly group was higher than that in the elderly group (*P* < 0.01). Furthermore, the incidence of incision complications in the elderly group was higher than in the non‐elderly group (14.29% > 1.78%, *P* < 0.01). The serum PA level was graded in accordance with NRS2002. There were 88 patients with preoperative grade 0–1 serum PA level (≥0.16g/L) who had no incision complications. The incidence of incision complications in patients with grade 3 serum PA level (<0.10 g/L, 9 patients) was higher than in patients with grade 2 (0.100–0.159 g/L, 66 patients) (44.44% > 6.06%, *P* < 0.01).

**Conclusion:**

Changes in serum PA level in patients with spinal tuberculosis during the perioperative period are consistent with the trend of inflammation control and nutrition improvement, and are correlated with the incidence of incision complications after surgery. The relationship between the changes and the timing of surgery is worthy of future research.

## Graphical abstract

Preoperative evaluation of spinal tuberculosis Prealbumin (PA): a balanced indicator for infection and nutrition

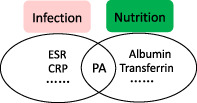

## Introduction

Nearly one‐third of the human population is infected with tuberculosis (TB). Of those with active disease, approximately 10% are impacted by skeletal tuberculosis^1^. Spinal TB is one of the most dangerous forms of skeletal TB and accounts for 50% of all skeletal TB^2^.

The cornerstone of medical management is multidrug chemotherapy to minimize relapse and drug resistance, and surgical management is reserved for patients presenting with neurological deficits or severe kyphosis. The aims of surgery are to treat the neurological deficits, correct the kyphotic deformities, and achieve and maintain stability. With appropriate and timely management, clinical outcomes of the treatment for spinal tuberculosis are excellent overall.

With the progress in surgical technology and implants, under the protection of anti‐tuberculosis drugs, the safety and effectiveness of internal fixation for patients with spinal tuberculosis accompanied by surgical indications have been widely recognized, but the timing of surgery remains controversial^3^. Inappropriate surgical timing is related to complications after spinal tuberculosis surgery, such as intestinal obstruction, postoperative stress response, sinus formation, wound infection and nonunion, bone graft nonfusion, and internal fixation loosening^4^, ^5^.

At present, it is unanimously acknowledged that adequate preoperative anti‐tuberculosis treatment can reduce the number of tubercle bacilli in lesions, inhibit the growth of tubercle bacilli, and avoid the whole‐body transmission of tubercle bacilli caused by surgery. Preoperative application of anti‐tuberculosis drugs for 2–4 weeks^6^, ^7^ is a method accepted by many surgeons. Some studies have detected the DNA content of tubercle bacilli in peripheral blood by polymerase chain reaction (PCR), and have found that for patients with spinal tuberculosis, whose anti‐tuberculosis treatment is <2 weeks, and whose erythrocyte sedimentation rate (ESR) has not decreased to the normal level, surgery has no significant effect on the transmission of tubercle bacilli *in vivo* ^8^, ^9^; but these studies have not changed the current clinical choice.

The ESR is the criterion used by many surgeons in the selection of the timing of surgery, but it has been questioned. The reduction of preoperative ESR to a specific delimited value, such as <50 mm/1 h or <40 mm/1 h^5^, or even to a normal ESR, is considered the indication for surgery. The change in ESR is related to the severity of tuberculosis and the immune status of the body. An abnormal immune status may mean that the ESR of patients with tuberculosis does not fully reflect the activity of tuberculosis and the severity of the disease^10^. It is often clinically observed that the increase in ESR in patients with tuberculosis is inconsistent with the poisoning symptoms of tuberculosis. Placing all attention on ESR while neglecting the changes in the symptoms, signs, and imaging data may lead to deductive errors. Some studies have shown no obvious relationship between preoperative ESR and the postoperative recurrence rate in spinal tuberculosis, and have concluded that ESR cannot be used as a key criterion for surgery^11^. Other studies have revealed that ESR is not particularly valuable in determining the activity of tuberculosis, and that C‐reactive protein is more sensitive, can provide an early reflection of the changes in the disease, and is more valuable in determining the activity of tuberculosis^12^. However, C‐reactive protein has high sensitivity but poor specificity in determining the inflammation activity. If it is used alone as a reference for surgery, it may overestimate the severity of inflammation and delay the timing of surgery.

For elderly patients with spinal tuberculosis, choosing the timing of surgery is somewhat a challenge. The incidence of spinal tuberculosis in the elderly is increasing year by year. Elderly patients often have diabetes, hypertension, chronic bronchitis, and other chronic diseases. They also often have a reduced tolerance to surgery and high risk of surgery^13^. Kyphosis‐induced deformity caused by bone destruction in the elderly develops rapidly, and the spinal nerve function is easily damaged. Being long‐term bedridden can also lead to a series of complications, and the degree of damage may sometimes exceed that of spinal tuberculosis itself. Therefore, early diagnosis is important and surgery should be performed for elderly patients with spinal tuberculosis^14^. However, the immunity of the elderly can be weak, the poisoning symptoms of tuberculosis, such as fever and night sweats, are not always obvious, and ESR and C‐reactive protein may not increase. Diagnosis can be missed in the early stages. It is also difficult to determine the effectiveness of preoperative anti‐tuberculosis treatment. Moreover, the control of medical complications affects the timing of surgery.

There is a two‐way relationship between tuberculosis and nutrition. Tuberculosis can lead to nutritional risk and patients are prone to nutrition‐related diseases, such as malnutrition, drug‐induced liver injury, low immune function, pulmonary infection, and electrolyte disturbance, which increase the risk of failure of anti‐tuberculosis drug treatment and delay focus repair. Patients with spinal tuberculosis are prone to weight loss and appetite decline in the early stage of tuberculosis, and their nutritional risk score tends to be high at admission. Correcting malnutrition can improve the body's negative nitrogen balance, enhance immunity, ensure the effective bactericidal concentration of anti‐tuberculosis drugs after surgery, and maintain the metabolism of cells, organs, and tissues so that they can function normally, accelerate tissue repair, and promote the rehabilitation of patients^15^.

The incidence of complications after spinal tuberculosis surgery is affected by many factors, among which the control of tuberculosis infection and nutritional status are two critical factors. However, there are still no reliable indicators available to guide the timing of surgery^4^, ^13^. Serum prealbumin (PA) not only reflects the control of inflammation but also serves as a sensitive indicator of nutritional status. The trend of changes in the perioperative period of spinal tuberculosis and its relationship with the complications after surgery have not been studied. In this paper, a preliminary study was conducted focusing on the relationship between changes in PA level and incision complications after surgery in patients with spinal tuberculosis during the perioperative period, so as to explore reliable indicators for guiding the timing of the spinal tuberculosis surgery. Few studies have reported the perioperative trend of spinal tuberculosis and its relationship with postoperative complications. Therefore, this retrospective study examines the changing trend of the serum PA level in patients with spinal tuberculosis during the perioperative period, and discusses the relationship between the PA level and postoperative incision complications. The purpose of this study is: (i) to summarize and analyze the clinical changes of the serum PA level during the perioperative period; (ii) to reveal the correlation between the PA level and the incidence of postoperative incision complications; (iii) to explore the clinical significance of PA as an index reflecting inflammation control, nutritional status, and immune status.

## Patients and Methods

### *Patient Demographics*

A retrospective study was performed on 162 patients (82 men and 80 women) with spinal tuberculosis who were admitted to our hospital from June 2013 to June 2017.

These patients were then assigned to the elderly group (≥65 years of age, *n* = 35) and the non‐elderly group (<65 years of age, *n* = 127). The inclusion criteria followed the PICOS principle: (i) confirmed by imaging, histopathology, and bacteriology, the diagnosis is active spinal tuberculosis; (ii) after regular antituberculosis treatment, all patients have surgical indications and received primary debridement and/or bone graft fusion and internal fixation for spinal tuberculosis; (iii) the experimental subgroups reported in the article include the elderly group and the non‐elderly group; (iv) the related outcomes of patients were recorded; and (v) the study design was a retrospective study. The exclusion criteria for this study are as follows: (i) age <17 years; (ii) the focus specimen is confirmed to be a suppurative infection caused by other bacteria; (iii) patients were excluded with the diagnosis of other diseases related to malnutrition, such as chronic diarrhea, liver cirrhosis, and malabsorption syndrome, and other diseases related to inflammatory immunity, such as rheumatoid arthritis, ankylosing spondylitis, and lupus erythematosus.

The composite reference standard (CRS) refers to the guidelines for the diagnosis of bone and joint tuberculosis in the tuberculosis diagnostic guidelines^16^ developed by the National Health and Clinical Optimization Agency (NICE) in 2011. The diagnostic criteria for spinal tuberculosis in this study are: (i) the symptoms and signs are consistent with the abscess and dead bone manifestations of local tuberculosis lesions found by imaging (including X‐ray, CT, and MRI examinations); (ii) pathological examination of the focus specimen showed caseous necrosis and Langerhans granuloma; (iii) microbiological examination of focus specimens (traditional Roche culture) confirmed *Mycobacterium tuberculosis* infection; and (iv) after regular antituberculosis drug treatment for more than 6 months, the symptoms of tuberculosis poisoning and local symptoms disappeared. Spinal tuberculosis is diagnosed if any two of the above criteria are met.

### *Perioperative Management*

Included patients were provided with both nutritional support and regular anti‐tuberculosis chemotherapy regimen 3HREZ/9HRE after admission. After 3 to 4 weeks of treatment, the systemic symptoms of tuberculosis as well as anemia and hypoalbuminemia were improved, and surgery was conducted when ESR < 50 mm/1 h or C‐reactive protein (CRP) < 30 mg/L. Postoperative drainage, application of anti‐tuberculosis therapy, and nutritional support were provided. The body temperature of patients was normal for more than 3 days in duration at discharge; the incisions were healed and the sutures were removed; posterior and lateral radiographs of the spine and chest X‐ray showed normal changes after surgery; B‐ultrasound of the psoas muscle showed little fluid in residual cavity.

### *Clinical Index Observation*

The serum PA levels at admission, prior to surgery, and at 2 and 4 weeks after surgery were measured by transmission immunoturbidimetry (reagents provided by Ningbo Medical System) using a Toshiba TBA‐120FR biochemical analyzer. The normal value of serum PA was 0.15–0.4 g/L. Follow up of 3 months was performed to observe incision healing and sinus formation in the included patents. The serum PA level was graded in accordance with NRS2002: grade 0–1 serum PA level (≥0.16 g/L), grade 2 (0.100–0.159 g/L), and grade 3 serum PA level (<0.10 g).

### *Statistical Analysis*

The data were expressed as mean ± standard deviation, and statistical processing was performed using SPSS 22.0 software. Changes in serum PA levels of all patients at different time points were observed using one‐way analysis of variance. Pairwise comparison at different time points was performed using the least significant difference method, and comparison of serum PA levels between different groups at the same time points (elderly group and non‐elderly group) was subjected to *t*‐test. The χ^2^‐test was used for comparison of the incidence of incision complications between the elderly group and the non‐elderly group and between different subgroups based on different PA levels. *P* < 0.05 was considered statistically significant.

## Results

### *Changes in Serum Prealbumin Levels*

There was a gradual increasing trend in the level of serum PA from admission to 4 weeks after surgeryin all 162 patients with spinal tuberculosis [(0.14 ± 0.03) g/L < (0.16 ± 0.04) g/L < (0.22 ± 0.04) g/L < (0.25 ± 0.04) g/L]. The trend of changes in serum PA levels in the non‐elderly and elderly groups was consistent with that of all subjects, and the elevation in the non‐elderly group was higher than that of the elderly group (*P* < 0.01; Table 1).

**TABLE 1:** Trend of changes in serum prealbumin level in patients with spinal tuberculosis during the perioperative period

Groups	Admission	Prior to surgery	2 weeks following surgery	4 weeks following surgery	*P*
Elderly group	0.12 ± 0.04*	0.13 ± 0.04*	0.18 ± 0.05	0.23 ± 0.05	0.000
Non‐elderly group	0.14 ± 0.03	0.17 ± 0.03	0.23 ± 0.03	0.25 ± 0.03	0.000
Overall	0.14 ± 0.03	0.16 ± 0.04	0.22 ± 0.04	0.25 ± 0.04	0.000
*P*	0.000	0.000	0.000	0.002	

* Pairwise comparisons showed no statistically significant difference.

### *Incidence of Incision Complications*

The incidence of incision complications was higher in the elderly group than in the non‐elderly group (14.29% > 1.78%), and the difference was statistically significant (*P* < 0.01). The serum PA level was further graded in accordance with NRS2002. There were 88 patients with grade 0–1 serum PA level (≥0.16 g/L) prior to surgery who had no incision complications, and the incidence of incision complications in patients with grade 3 serum PA level (<0.10 g/L, 9 patients) was higher than that in patients with grade 2 (0.100–0.159 g/L, 66 patients) (44.44% > 6.06%), with the difference being statistically significant (*P* < 0.01), as shown in Tables 2 and 3.

**TABLE 2:** Incidence of incision complications in subgroups based on age

Groups	Normal	Abnormal	Incidence of incision complications (%)	χ^2^	*P*
Elderly group	30	5	14.29	9.168	0.002
Non‐elderly group	169	3	1.78		

**TABLE 3:** Incidence of incision complications in subgroups based on serum PA levels

Albumin level	Normal	Abnormal	Incidence of incision complications (%)	χ^2^	*P*
Grade 3	5	4	44.44	8.549	0.003
Grade 2	62	4	6.06		

## Discussion

### *Prealbumin Level and Liver Function, Inflammatory State, and Immune State*

As a plasma protein synthesized by hepatocytes, serum PA has a short biological half‐life (1.9 days). Its main physiological functions are to transport thyroxine and vitamin A, and enhance the immunity of the body by promoting maturation of lymphocytes. It shows good sensitivity and specificity profiles for hepatocyte inj ury^17^, and is more sensitive than serum albumin, for which it can serve as a useful indicator for monitoring of nutritional assessment and nutritional support^18^. Simultaneously, serum PA level is negatively correlated with the that of infections and immune inflammation of the body. The possible mechanisms may be that the inflammatory mediators injure the hepatocytes and hepatic sinusoidal endothelial cells, resulting in ischemic and hypoxic tissues and organs, fibrotic liver tissues, and, thereby, reduced PA synthesis^19^, ^20^. Patients with spinal tuberculosis have reduced nutrient intake, progressed infections, and inflammatory immune responses.

In this study, we observed the changes in serum PA level during the perioperative period. With anti‐tuberculosis drug treatment and nutritional support, the serum PA level gradually rose and further increased after surgery, which was consistent with the decrease of inflammation and nutrition improvement in the patients. Furthermore, the serum PA level was lower in the elderly group than in the non‐elderly group, which was consistent with the age‐associated decline in immune and nutritional status. It is speculated that the serum PA level can satisfactorily reflect the liver function, inflammations, and the immune status of patients, for which further explorations are merited.

### *Prealbumin Level and Postoperative Incision Complications, and Operation Timing*

The incidence of complications after spinal tuberculosis surgery is affected by many factors, among which the treatment for tuberculosis and nutritional status occupy an important position. However, there are no reliable indicators available to guide the timing of surgery. Currently, the number of elderly patients with spinal tuberculosis is increasing gradually, many of whom have other concomitant diseases and diminished nutritional and immunomodulatoryfunction^21^, ^22^. Symptoms of tuberculosis such as fever and night sweats are typically not obvious, and the ESR and CRP are often infected by many factors, which restrict the observation of tuberculosis control truly and intuitively. Previously, timing of surgery was selected on the basis of preoperative systemic and local symptoms, nutrition, hematology, ESR and CRP.

In fact, it is more necessary to strengthen the preoperative nutritional support and anti‐tuberculosis drugs for elderly patients with multiple diseases related to nutrition and immunity, and surgical treatment should be provided as soon as possible based on sensitive indicators. Serum PA levels not only reflect the control of infections but also serve as a sensitive indicator of nutritional status. In this study, the score of the elderly group was higher than that of the non‐elderly group (3.49 ± 1.62 > 2.61 ± 1.39) according to the NRS2002 Nutritional Assessment Scale, and the incidence of incision complications was higher in the elderly group than that in the non‐elderly group. The serum PA level was further graded based on the NRS2002 score, and the incidence of incision complications in patients with grade 3 (<0.10 g/L) was higher than that in patients with grade 2 (0.100–0.159 g/L). It is speculated that the serum PA level can serve as an evaluation indicator before surgery to guide the timing of surgery, which merits further explorations.

### *Limitations*

The limitations of this study include the small number of patients and the short‐to‐medium follow‐up. In addition, this is only a single‐center study in our hospital. In the later stage, we need to conduct multi‐center case studies with more cases and long‐term follow‐up for further observation.

## Conclusion

Changes in serum PA level in patients with spinal tuberculosis during the perioperative period are consistent with the trend of control of inflammations and nutrition improvement, and correlate with the incidence of incision complications after surgery. Its relationship with the timing of surgery should be examined further.
